# Lack of Gender-Disaggregated Data for the Psychological Impact of ACL Injury on Adolescents

**DOI:** 10.3390/jcm14144885

**Published:** 2025-07-09

**Authors:** David Holdroyd, Benjamin Gompels, Ilias Epanomeritakis, Alexandra Macmillan, Simone Castagno, Hans Johnson, Stephen McDonnell

**Affiliations:** 1 Department of Trauma and Orthopaedics, Addenbrooke’s Hospital, University of Cambridge, Hills Rd, Cambridge CB2 0QQ, UK; bdg30@cam.ac.uk (B.G.); iee21@cam.ac.uk (I.E.); am710@cam.ac.uk (A.M.); sc2257@cam.ac.uk (S.C.); sm2089@cam.ac.uk (S.M.); 2 University Hospitals Bristol and Weston, Marlborough Street, Bristol BS1 3NU, UK; hj14789@bristol.ac.uk

**Keywords:** anterior cruciate ligament, adolescent, psychology, depression, anxiety

## Abstract

**Background**: This literature review examines the prevalence and severity of depression and anxiety symptoms in adolescents following ACL injury, with a focus on gender-disaggregated data. **Methods**: An electronic search was conducted across databases, including Ovid MEDLINE (R), PUBMED, and the Cochrane Library, covering articles from 1964 to 13 February 2024. Primary search terms were “ACL,” “depression,” “anxiety,” and “adolescent,” expanded using medical subject headings (MeSH). Manual searches of the references supplemented the database search. Inclusion criteria included studies on depression or anxiety post-ACL injury in adolescents. The exclusion criteria were studies without adolescent participants or those focusing exclusively on one sex. **Results**: The search identified 62 studies, of which 5 were ultimately selected for comprehensive analysis. Four studies reported on depression using various scales, and three measured anxiety symptoms. No studies provided gender-disaggregated data. One study found no significant difference in depression scores between adolescent and adult patients. Another study noted that socioeconomic disadvantage correlated with increased depressive and anxiety symptoms post-surgery. An additional study highlighted a significant increase in depression scores from pre- to post-injury among ACL-injured participants compared to uninjured controls. **Conclusions**: Our understanding of the psychological consequences of ACL rupture on female adolescent patients is incomplete. Given the high incidence of ACL injuries in this patient group and their potential psychological vulnerability, improving the evidence base in this area could address a previously neglected aspect of care, with positive impacts on returning to sport and quality of life. Future studies of high methodological quality are needed to address this gap in the literature.

## 1. Introduction

The anterior cruciate ligament (ACL) is the most injured soft tissue structure in the knee joint [1,2]. The incidence of ACL injuries is 71 per 100,000 individuals per year, and this is increasing, especially among the paediatric population, with a 143% increase in ACL injuries reported among girls aged 13 to 15 over 18 years [3,4]. The surge in ACL injury incidence aligns with a corresponding increase in the UK’s surgical treatment of soft tissue knee injuries (STKI). Approximately 15,000 ACL reconstructions are performed annually, reflecting a substantial growth in these procedures over the past two decades, with estimates suggesting a twelvefold increase in the UK [5,6]. ACL injury can present a significant economic health burden. The long-term sequelae of ACL injury can include secondary osteoarthritis, observed in up to 50–90% of individuals, and the risk of re-injury seen in up to one in five individuals presenting with an ACL injury [7].

The diagnosis and treatment of these injuries are constantly evolving areas of research and practice. The early diagnosis of such injuries has benefited from both advances in clinical tests and more advanced imaging modalities. The use of the lever test has emerged as a relatively new technique for clinically diagnosing ACL injuries, showing reliable specificity and sensitivity [8,9]. With MRI availability often posing a strain on health services, the use of point-of-care ultrasound has recently demonstrated promise as an additional tool for the early confirmation of complete ACL rupture [10]. Despite this, it should be noted that MRI remains the gold standard for imaging, particularly in the context of associated injuries that could otherwise be missed [11].

Concurrently, the prevalence of mental health issues in young people has increased, with one in five young people aged 8 to 25 being reported to have a probable mental health disorder in the UK in 2023. In addition to the detriment to the quality of life of the individuals affected, this also constitutes a significant burden to the NHS. The overall rates of mental health disorders are similar between genders between the ages of 8 and 16, whilst in those aged 17–25, the prevalence is twice as high in females. Despite the similar rates in younger age groups, the manifestations of such disorders differ. For example, females between 11 and 16 are four times more likely to develop an eating disorder in comparison to males of the same age [12].

ACL injury is known to be associated with a negative psychological impact, which is multifactorial. Pain following an injury has been linked with exacerbated poor mental health [13]. In addition, the importance of physical activity as a protective factor for mental health issues is well documented [14]. However, the extended period in which patients are unable to participate in their chosen sport due to ACL injury compounds the effect on patients’ mental health [15,16,17]. This is particularly important to consider as poor psychological outcomes following ACL reconstruction have been linked to inferior rehabilitation outcomes and, therefore, a reduced likelihood of returning to pre-injury sporting levels [18,19,20]. The literature has described the associated negative psychological impacts during the rehabilitation period [21]. This includes symptoms of psychiatric disorders such as depression and anxiety, with depression showing an increased incidence following a musculoskeletal injury in comparison to the general population [22,23].

The convergence of the increased vulnerability of adolescents, especially those who are of the female sex, and suffering an ACL injury, combined with their increased likelihood of developing depression or anxiety, highlights the importance of an integrated, holistic, patient-centred approach to managing these injuries. This literature review examines the available literature reporting the prevalence and severity of symptoms of depression and anxiety in adolescents following an ACL injury, as well as looking at the availability of gender-disaggregated data.

## 2. Materials and Methods

### 2.1. Search Strategy

An electronic search was conducted across several databases, including Ovid MEDLINE (PUBMED) and the Cochrane Library. The search was performed by two authors, DH and BG, who independently searched articles published from 1964 to 13 February 2024. The primary search terms used were “ACL”, “depression”, “anxiety”, and “adolescent”. These terms, along with related keywords, were expanded using medical subject headings (MeSH) and strategically combined using Boolean operators (“AND”, “OR”) with associated keywords. Additionally, a manual search was conducted to review the reference lists of the identified articles, aiming to identify relevant studies that may not have been indexed in the searched databases.

### 2.2. Inclusion and Exclusion Criteria

Authors DH and BG independently screened abstracts against the inclusion criteria. If insufficient information was provided in the abstract to decide, the search was advanced to a full-text screening alongside other abstracts that met the initial screening criteria. Any disagreement was resolved by a third reviewer (SC). Studies were included if they presented original primary research findings on depression or anxiety post-ACL injury and/or reconstruction among adolescents. Only articles available in the English language were considered. Exclusion parameters were as follows: (1) absence of adolescent participants aged 12–18; (2) studies focusing exclusively on one gender.

## 3. Results

**Figure 1:**
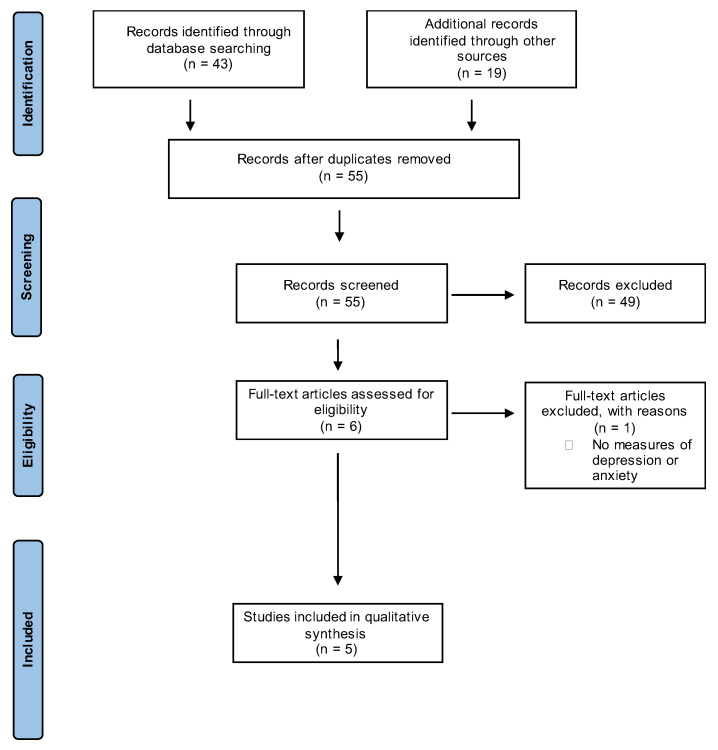
PRISMA flow chart showing the search strategy.

In the initial search phase, we identified 62 studies, comprising 43 from database searches and 19 from additional sources, such as hand-searching. Subsequent reference checks revealed a further 19 articles. Seven duplicative studies were eliminated. During the initial review of titles and abstracts, 49 studies were dismissed based on predetermined exclusion criteria. Following an in-depth analysis of the full texts of the six remaining articles, one further study was excluded. Ultimately, five studies were selected for comprehensive analysis. The process of identification and selection is illustrated in Figure 1. As shown in Table 1, the quality assessment scores were assessed using the Newcastle–Ottawa Score (NOS) [24] for non-randomised studies [25,26,27,28] and the Jadad scale for the randomised studies [29,30]. The NOS uses a star system, with a maximum of nine stars to score non-randomised studies across three domains: the selection of study groups (maximum four stars), the comparability of study groups (maximum two stars), and the ascertainment of the outcome (maximum three stars). Eight to nine stars is considered very good quality, six to seven as good quality, four to six as satisfactory, and three or less as unsatisfactory [31]. Based on this, two papers scored as good quality [27,28] and two as satisfactory quality [25,26]. The Jadad scale was used for randomised studies scores across three items—randomisation, blinding, and dropouts or withdrawals––with a maximum score of five. The one included randomised study scored 3 points, which is considered to be indicative of a high-quality study [32]. The articles encompassed a publication period from 2003 to 2023, with only two being published within the last decade.

**Quality assessment**

**Table 1:** A summary table presenting the quality assessment scores of the papers included for qualitative synthesis.

Lead Author (Year)	Quality Assessment Score (Quality Assessment Tool)
Tripp et al., 2003 [25]	5 *: Selection 3, Comparability 0, Outcome 2 (NOS)
Baranoff et al., 2015 [26]	5 *: Selection 2, Comparability 1, Outcome 2 (NOS)
Mainwaring et al., 2010 [27]	6 *: Selection 4, Comparability 0, Outcome 2 (NOS)
Kiani et al., 2023 [28]	6 *: Selection 4, Comparability 0, Outcome 2 (NOS)
Maddison et al., 2006 [29]	3/5 (Jadad scale)

NOS: Newcastle–Ottawa Scale. *: Number of stars awarded to each paper using Newcastle-Ottawa Scale.

### 3.1. Depression

Four out of the five included studies recorded symptoms of depression as an outcome variable, with all using different measurement scales. These included the Beck Depression Inventory (BDI) [25], a 21-question self-report questionnaire which acts as a measure of depression severity and is validated for the adolescent population [33]. The Depression Anxiety and Stress Scale (DASS-21) [26] was also used, which is a combination of three self-report scales that report the severity of measures of depression, anxiety, and stress. This has also been validated for use in adolescents [34]. The Profile of Mood States (POMS) [27] comprises 65 items to assess individuals across seven mood domains; however, it has not been validated for adolescents. POMS-A was developed [35], which is recommended and validated for use in this group. The Patient-Reported Outcomes Measurement Information System (PROMIS) [28] assesses a range of health measures encompassing physical, mental, and social health. It was demonstrated to be valid and reliable in adolescent populations [36]. As shown in Table 2, the timescales over which data were collected were variable. One paper only recorded data at a single time point [25]. One paper collected data at the pre-operative appointment and again at a 6-month post-operative assessment [26]. Another paper collected data at multiple time points within the first month of injury [27]. One paper used multiple time points over a minimum of 6 months.

**Depression**

**Table 2:** A summary of the studies that investigated the correlation between anterior cruciate ligament (ACL) injury and depressive symptoms.

Lead Author (Year)	Population	Age of Participants	Gender Split	Baseline Measurement	Follow Up Time	Outcome Variable	Findings at Baseline, Type of Patient, Outcome Values: Mean ± SD	Findings at Follow-Up	Gender Regression or Disaggregation	Age Regression or Disaggregation
Tripp et al., 2003 [25]	30 patients (20 adults, 10 adolescent)	Adolescent: 16–18, Adult: 20–53	Adolescent: 5 female/5 male Adult: 4 female/6 male	1 day post-operatively	NA	BDI	Adults: 7 ± 6.4 Adolescents: 10 ± 4.3 No significant difference between adolescent/adult groups for anxiety or depressive symptoms	NA	No	Yes
Baranoff et al., 2015 [26]	44 patients	Mean 27, SD 9.4	17 female/27 male	Within 14 days post-operatively	6 months post-operative	DASS 21	Baseline: 8.6 (7.4) No correlation of age/ gender with depressive symptoms.	6 months: 11 (11.6)	Regression	Regression
Mainwaring et al., 2010 [27]	51 participants (16 concussion, 7 ACL injury, 28 uninjured)	Mean 21.2, SD 2.94, range 17.5–37	30 female/21 male	Pre-injury (start of sporting season)	1 month (29 days) post-injury	POMS	ACL: 0.71 ± 0.95 Concussion: 1.31 ± 2.55 Control: 1.54 ± 3.13 No significant difference between groups at baseline	First post injury check: ACL: 5.00 ± 4.40 Concussion: 3.75 ± 4.55 Control: 1.54 ± 2.41 ACL group significantly different to control (*p* = 0.005) Significant main effect of time post-injury on depressive symptoms in ACL injury group	No	No
Kiani et al., 2023 [28]	413 patients	Median 15, IQR 13–16	223 female/ 190 male	5–54 days post-injury	9.1–12.6 months post-injury	PROMIS	No significant correlation of depressive symptoms with socioeconomic disadvantage at baseline	Depressive symptoms correlated with socioeconomic disadvantage at multiple post-operative time points	No	No

BDI: Beck Depression Inventory; DASS 21: Depression Anxiety Stress Scales—21 items; POMS: Profile of Mood States; PROMIS: Patient-Reported Outcomes Measurement Information System.

Only one of the included studies used age-disaggregated data, comparing the depressive symptoms of adolescent and adult patients during their post-operative assessment [25]. This study did not find a significant difference in depression scores between adolescent and adult groups. However, the sample size was small (10 adolescents and 10 adults), and these scores were only measured 24 h post-operatively, which may not have been enough time for depressive symptoms to precipitate. Additionally, this paper did find that adolescents showed greater catastrophising, helplessness, and rumination-based catastrophic thought, factors which have been shown to relate to increased emotional distress [37]. Another paper included a regression analysis demonstrating that age did not correlate with the depression outcome variable [26]. This paper also found no correlation between depressive symptoms and gender, a factor which was not reported in any of the other studies. Even in this paper, the specific data for these variables were not reported. None of the included studies included gender-disaggregated data.

One of the studies investigated the relationship between depressive symptoms and social determinants of health over time following ACL injury [28]. Interestingly, at baseline, there was no significant correlation of depressive symptoms with socioeconomic disadvantage. However, a significant correlation was observed at multiple post-operative time points. This demonstrates how differential vulnerabilities to the development of depressive symptoms may be precipitated by stressors such as ACL injury. The lack of gender disaggregation in these studies, unfortunately, misses an opportunity to examine whether this increased vulnerability impacts patients disparately according to their gender. This delay in differential findings between groups may provide insight into the results of the study, which found no difference in depression scores between adults and adolescents 24 h post-operatively [25].

A comparison between pre- and post-injury depressive scores was included in one of the studies [27]. The change in depressive symptoms between the baseline assessment and the first post-injury assessment in participants who had sustained an ACL injury differed significantly from that of an uninjured control group. This study also demonstrated an increase in depression scores of more than seven times between these time points in the ACL injury group. This was a more significant increase than was seen in an additional group who had suffered concussions.

### 3.2. Anxiety

Three of the included studies included measurement of symptoms of anxiety following ACL injury, as seen in Table 3. Two papers [25,29] used the State-Trait Anxiety Inventory (STAI-S), a self-reporting questionnaire which measures both the patient’s current ‘state’ anxiety levels as well as their general, ‘trait’, anxiety levels, and is validated for use in adolescents [38]. The other used the PROMIS [28]. One of these studies disaggregated the results of the adult and adolescent populations [25]. The increase in anxiety symptoms in the teenage group was not found to be significant. Again, this was only recorded at the 24 h post-operative stage. None of the included papers disaggregated the results by participants’ gender. As with depression scores, the paper investigating the association between socioeconomic group and anxiety symptoms [28] found a correlation at multiple post-operative time points but not at baseline. This suggests that socioeconomic status interacts similarly with both depression and anxiety symptoms following ACL injury. One paper [29] examined the effect of a modelling intervention on anxiety symptoms. This paper demonstrated an increase in anxiety symptoms from baseline to pre-operative assessment in both groups, but no significant difference between the groups. However, the modelling intervention was successful in reducing expected pain pre-operatively. The modelling condition group showed a reduction in expected pain from baseline to the pre-operative assessment whilst the control group showed an increase in expected pain across this period. This study only collected data relating to anxiety at baseline and during pre-operative assessments. This limits the inferences that can be drawn from this study as it is unclear whether the modelling intervention may have had longer-term benefits in the recovery and rehabilitative period.

**Anxiety**

**Table 3:** A summary of the three papers that examined anxiety symptoms in patient cohorts post-ACL injury.

Lead Author (Year)	Population	Age of Participants	Gender Split	Baseline Measurement	Follow Up Time	Outcome Variable	Findings at Baseline, Type of Patient, and Outcome Values: Mean ± SD	Findings at Follow-Up	Gender Regression or Disaggregation	Age Regression or Disaggregation
Tripp et al., 2003 [25]	30 patients (20 adults, 10 adolescent)	Adolescent: 16–18, Adult: 20–53)	Adolescent: 5 female/5 male. Adult: 4 female/6 male	24 hrs post-operatively	NA	STAI-S	Adults: 33.80 ± 8.78 Adolescents: 44.40 ± 12.43. No significant difference between groups for anxiety symptoms	NA	No	Yes
Maddison et al., 2006 [29]	58 patients. (30 modelling group, 28 control group)	15 to 53	32% female, 68% male	First assessment	Pre-operative assessment	STAI-S	Intervention: 36.93 ± 8.85 Control: 34.25 ± 7.80 No significant difference between groups	Intervention: 38.17 ± 10.19 Control: 37.52 ± 6.52 No significant difference between groups General increase in anxiety symptoms from baseline to pre-operative assessment in both groups	No	No
Kiani et al., 2023 [28]	413 patients	Median 15, IQR 13–16	54% female/ 46% male	5–54 days post-injury	9.1–12.6 months post-injury	PROMIS	No significant correlation of anxiety symptoms with socioeconomic disadvantage at baseline	Anxiety symptoms correlated with socioeconomic disadvantage at multiple post-operative time points	No	No

STAI-S: State-Trait Anxiety Inventory-State version; PROMIS: Patient-Reported Outcomes Measurement Information System.

### 3.3. Summary

Overall, whilst some demographic factors, such as age and socioeconomic group, have been studied in terms of their relationship to depression and anxiety symptoms, none of the included papers provided gender-disaggregated data. The omission of such analysis acts as a barrier to identifying a potentially important risk factor for adverse mental health outcomes following ACL injury. By including such data, these papers may have provided insight not only into how gender may independently predict patient-reported outcomes but also into how it may interact with other demographic factors, which have been shown to increase a patient’s vulnerability.

One paper [28] demonstrated how increased impacts on mental health due to demographic factors may not occur in the acute setting, but may precipitate later on. This should be taken into account in future research by ensuring that later follow-ups are included, so that meaningful interactions are less likely to be missed.

## 4. Discussion

The results of this review demonstrate a gap in the literature regarding gender-disaggregated reporting of the psychological impacts of ACL injury in adolescent patients. However, age- and sex-specific factors are essential for tailoring care and should not be ignored.

The lack of gender disaggregation in the field may be due to several reasons. Historical research biases have often been underpinned by assumptions that findings in one group apply universally, with men frequently serving as the default [39]. These biases may be compounded in this field by the underrepresentation of women in trauma and orthopaedics. In the UK, women make up only 7.3% of trauma and orthopaedic consultants [40], despite having reached parity in medicine [41]. Whilst most research now records the sex of participants, it is often not analysed separately, potentially due to a perceived lack of statistical power for subgroup analysis or a continued assumption of homogeneity between groups. Improved cross-speciality collaboration between psychology and orthopaedics may benefit clinical outcome measures in both areas.

ACL rupture is a common sporting injury, and surgical intervention represents a growing proportion of the orthopaedic caseload [5,6]. Sporting injuries and mental health have a reciprocal influence on each other; both athletes and the general population suffer from a negative impact on mental health following injury, and poor mental health itself can increase injury risk [42,43]. Holistic care of knee injuries should involve addressing mental health consequences, which can impact engagement in rehabilitation, return to sporting activity, and broader quality of life [44,45].

The studies identified here commonly demonstrated an increase in depression and anxiety symptoms following ACL injury, a phenomenon which has already been described [21]. The purpose of our review was to identify existing evidence regarding the mental health consequences of ACL injury on female adolescents. Among the included studies, no gender-disaggregated data were reported to elucidate the differential impact on male and female athletes. However, the regression model used by one study did not find gender to be predictive of adverse mental health [26]. Regarding the effect of age, another paper found no significant difference in anxiety or depression indices between adolescent and adult patients [25]. The generalisability of both studies is limited due to the relatively small sample sizes. Other patient characteristics that were found to have a statistically significant negative impact on mental health following ACL injury included socioeconomic disadvantage [28] and associated concussion injury [27].

The temporal effect of the psychological impact of ACL injury on mental health has also been studied [21]. This systematic review demonstrated that whilst depressive symptoms were common following ACL injury, they were transient, with a common trend of an early peak followed by a decrease over two years. Despite this, recent qualitative work in the field has demonstrated that longer-lasting psychological impacts can be appreciated with broader consideration of the changes that ACL injury can impose on patients [46]. This study explored self-perceived changes in adolescents between 3 and 10 years following ACL reconstruction and identified 19 self-described ‘shifts’ that may occur during this time, which could have a negative, neutral, or positive impact on their return to sport and rehabilitation. These shifts included changes to motivation, expectations, confidence, athlete identity and mental health. Further research to elucidate how specific factors, including gender, influence these shifts could provide a basis for implementing targeted interventions to facilitate positive shifts and mitigate the negative impacts. Additionally, further qualitative work specifically related to female adolescents may provide valuable insights into the unique effects and stressors affecting this patient group that quantitative scores may overlook.

As more women are participating in elite sports, it is increasingly recognised that the incidence of ACL injury is particularly high among female athletes compared to their male counterparts [47]. The reasons behind this are unclear, but it may be due to anatomical, hormonal, and psychological differences that alter injury risk and the recovery rate. The personalised management of these patients not only requires awareness of the biological implications of sex-specific differences but also a psychological perspective. The intricate relationship between sex and gender in the context of societal norms is an additional dimension that requires consideration to optimise post-injury outcomes [48].

Female athletes face specific psychological stressors that are less pervasive in the male sporting environment [49]. These can include increased vulnerability to body image concerns, pay disparities, the impact of family planning, and violence [49,50,51]. Following injury, depression and anxiety may compound pre-existing stressors, with the result being that female athletes are less likely to return to sport following injury [50]. The presence of such stressors should be considered when developing targeted interventional approaches to reach those most at risk.

The mental health of adolescent athletes following injury may also be underappreciated, given that most research in this area is conducted on the adult population [52]. Engagement in sporting activity is protective against psychiatric morbidity in adolescents, and time away from sport due to injury increases vulnerability to depression and anxiety. One study of 135 adolescents who suffered from ACL tears demonstrated an essential impact on self-esteem following injury and associated social limitations [53]. Given the close association between sporting activity and adolescent social development [52], it is unsurprising that a robust athletic identity is an independent risk factor for depression post-injury [54]. Research indicates that knee injuries can significantly impact knee function and quality of life for the first 12 months, particularly in adolescent females [55]. 

The included studies showed heterogeneity in their choice of measurement scale for depression and anxiety symptoms. The implementation of appropriate psychological support following injury will depend on the use of validated mental health indices. The UK’s National Institute for Health and Care Excellence (NICE) recommends screening for depression in adolescents using the Mood and Feelings Questionnaire (MFQ) [56], which has shown high diagnostic accuracy in adolescent populations [57]. This should be coupled with an individualised assessment of potential risk factors for depression, including acute life events [58]. Depending on an adolescent’s social background and reliance on sports as a social outlet, time away from sports due to injury can be considered a significant event. The common use of recommended tools for specific measures in this field would enhance the generalisability of findings across studies.

Additional assessments of mental state specific to sporting injuries may include fear of re-injury, which is reported at higher rates in female athletes [47] and has shown an association with poorer rehabilitation outcomes [58]. In a meta-analysis examining 48 studies of 5770 patients, fewer than half of the patients who underwent ACL reconstructions returned to competitive sports after a mean follow-up of 3.5 years, with fear of reinjury being the leading reason for reduced participation [59]. The identification of patients with a greater fear of re-injury following ACL injury may allow for them to receive targeted psychological support and ensure earlier engagement in the rehabilitation process [60]. Examples of recent advances in this area include technology-enabled support formats such as the “Back in the Game” app. Such advances serve as a low-cost psychological intervention that complements patients’ standard rehabilitation in a self-guided manner, potentially leading to improvements in their overall care.

The recovery period following ACL injury can also be influenced positively or negatively by an individual’s personality and attitude traits. For example, it has been shown that pre-surgery optimism can have a positive indirect effect on perceived knee function at 12 months post-surgery, as well as rehabilitation adherence [61]. Conversely, traits such as neuroticism have been shown to have a negative impact on recovery. Neuroticism has been shown to negatively influence subjective knee scores as well as more objective tests, such as the balance test in patients following ACL injury [62]. This is highly relevant to the consideration of the need for gender-specific research when considering the differing distributions of such personality traits across males and females. For example, it has been well-documented that women are more likely to score higher on measures of neuroticism [63,64].

Recent work studying the efficacy of psychological interventions following ACL injury demonstrated improved short-term outcomes for kinesiophobia and pain in comparison to standard rehabilitation [65], albeit with a low-quality evidence. By improving our understanding of which patient groups are most negatively psychologically affected by these injuries, as well as those that are most positively responsive to interventions, we can enhance the efficacy of such services.

Future assessments of the impact of ACL injury on female adolescent mental health should appropriately control for potential confounding factors. As previously mentioned, socioeconomic disadvantage and associated concussion injuries were independently associated with worse mental health outcomes following ACL injury [27,28]. Additionally, a comparison should be made with a group of uninjured peers or injured male controls.

## 5. Conclusions

Our understanding of the consequences of ACL rupture on the mental health of female adolescent patients is incomplete. Given the high incidence of ACL injuries in this patient group and their potential psychological vulnerability, improving the evidence base in this area could address a previously neglected aspect of care, with positive impacts on returning to sport and quality of life. There is potential for high heterogeneity and many confounding factors among studies investigating such outcomes. The lack of currently available literature in this area is a limitation of this paper. Further research into this field will provide a broader perspective so greater inference may be drawn.

Future studies of high methodological quality are needed to address the existing gap in the literature. Specifically, these should include prospective cohort studies to better understand the temporal variations in the psychological impact of ACL injuries. Such studies ought to comprise gender-disaggregated data as a primary outcome measure to clarify how these groups are differentially affected. Considering social factors, such as socioeconomic measures, may elucidate how these elements interact with gender and provide a foundation for a more equitable approach to care. Gender-disaggregated data should also be reported in interventional research, prioritising the development of targeted psychological interventions for female adolescent athletes, given their increased vulnerability to both ACL injuries and mental health issues.

## Abbreviations

The following abbreviations are used in this manuscript:

BDI	Beck Depression Inventory
DASS 21	Depression Anxiety Stress Scales—21 items
POMS	Profile of Mood States
PROMIS	Patient-Reported Outcomes Measurement Information System
STAI-S	State-Trait Anxiety Inventory-State version
NOS	Newcastle–Ottawa Score
